# Insulin Requirements and Carbohydrate to Insulin Ratio in Normal Weight, Overweight, and Obese Women With Type 1 Diabetes Under Pump Treatment During Pregnancy: A Lesson From Old Technologies

**DOI:** 10.3389/fendo.2021.610877

**Published:** 2021-02-25

**Authors:** Camilla Festa, Raffaella Fresa, Natalia Visalli, Olimpia Bitterman, Chiara Giuliani, Concetta Suraci, Marzia Bongiovanni, Angela Napoli

**Affiliations:** ^1^ Department of Clinical and Molecular Medicine Sapienza University of Rome, Rome, Italy; ^2^ Azienda Sanitaria Locale Salerno, Salerno, Italy; ^3^ Santo Spirito Hospital, Pescara, Italy; ^4^ Department of Experimental Medicine Sapienza University of Rome, Rome, Italy; ^5^ Policlinico Casilino, Rome, Italy; ^6^ Ospedale Israelitico, Rome, Italy

**Keywords:** obese BMI, obesity, continuous subcutaneous insulin infusion, gestation, type 1 diabetes, technologies, pregnancy management, overweight weight gain

## Abstract

**Aim:**

The primary aim of this study was to assess insulin requirements and carbohydrate to insulin ratio (CHO/IR) in normal weight, overweight, and obese pregnant women with type 1 diabetes across early, middle, and late pregnancy.

**Methods:**

In this multicenter, retrospective, observational study we evaluated 86 of 101 pregnant Caucasian women with type 1 diabetes under pump treatment. The women were trained to calculate CHO/IR daily by dividing CHO grams of every single meal by insulin units injected. Since the purpose of the study was to identify the CHO/IR able to reach the glycemic target, we only selected the CHO/IR obtained when glycemic values were at target. Statistics: SPSS 20.

**Results:**

We studied 45 normal weight, 31 overweight, and 10 obese women. Insulin requirements increased throughout pregnancy (p < 0.0001 and <0.001 respectively) in the normal and overweight women, while it remained unchanged in the obese women. Insulin requirements were different between groups when expressed as an absolute value, but not when adjusted for body weight. Breakfast CHO/IR decreased progressively throughout pregnancy in the normal weight women, from 13.3 (9.8–6.7) at the first stage of pregnancy to 6.2 (3.8–8.6) (p = 0.01) at the end stage, and in the overweight women from 8.5 (7.1–12.6) to 5.2 (4.0–8.1) (p = 0.001), while in the obese women it remained stable, moving from 6.0 (5.0–7.9) to 5.1 (4.1–7.4) (p = 0.7). Likewise, lunch and dinner CHO/IR decreased in the normal weight and overweight women (p < 0.03) and not in the obese women. The obese women gained less weight than the others, especially in early pregnancy when they even lost a median of 1.25 (−1 −1.1) kg (p = 0.005). In early pregnancy, we found a correlation between pregestational BMI and insulin requirements (IU/day) or CHO/IR at each meal (p < 0.001 and p = 0.001, respectively). In late pregnancy, a relationship between pre-gestational BMI and CHO/IR change was found (P = 0.004), as well as between weight gain and CHO/IR change (p=0.02). The significance was lost when both variables were included in the multiple regression analysis. There was no difference in pregnancy outcomes except for a higher pre-term delivery rate in the obese women.

**Conclusion:**

Pre-gestational BMI and weight gain may play a role in determining CHO/IR during pregnancy in women with type 1 diabetes under pump treatment.

## Background

Pregnant type 1 diabetic women have a high rate of obstetric and fetal complications such as preeclampsia, stillbirths, neonatal mortality, congenital malformations, and neonatal morbidity (1, 2).

A poor glycemic control during pregnancy, along with an inadequate pre-conceptional care, increases maternal–fetal complications (3, 4). Therefore, establishing correct insulin need during pregnancy is a key factor in order to prevent, on the one hand, the above-mentioned adverse outcomes, on the other, the risk of hypoglycemia.

Carbohydrate counting and carbohydrate-to-insulin ratio (CHO/IR) are a valuable tool in the management of type 1 diabetes in improving glycemic control and flexibility in eating habits (5, 6). Moreover, CHO/IR showed intra- and inter-individual variations which are not taken into account by most formulas, even more in pregnancy, when a number of metabolic factors change simultaneously and progressively.

Our previous observations confirmed a progressive CHO/IR decline over time at each meal in women with type 1 diabetes under continuous subcutaneous insulin infusion (CSII) therapy during pregnancy (7). Moreover, as pregnancy progressed, insulin requirements increased mainly due to a bolus dose increase (8–10).

In the last decades, all countries have been experiencing a dramatic rise in obesity due to several environmental factors, also affecting type 1 diabetic patients from childhood (11). Data from AMD annals (12) show that 23% of fertile Italian women with type 1 diabetes are overweight, and 7.8% are obese. Obesity increases insulin resistance even in type 1 diabetes (13). Because of the metabolic alterations during normal pregnancy, particularly the 60% decrease in insulin sensitivity, non-diabetic overweight and obese women are at increased risk of metabolic dysregulation in pregnancy (14). This pre-existing impairment should be considered in managing type 1 diabetic pregnancies in women with high pre-gestational BMI, since maternal overweight and obesity increase insulin resistance and insulin requirements especially during the second half of pregnancy (10, 15).

CSII use in pregnancy is still debated, and results obtained from the largest European RCT (16) point to a wide gap in the practice and experience with this technology in pregnancy.

Our hypothesis is that pre-gestational BMI class and weight gain may influence the CHO/IR trend throughout pregnancy. A deeper understanding of the role of these two variables may contribute to improving the accuracy of algorithms used as prediction models in new technologies.

### Aim

The aim of this retrospective study was to assess insulin requirements and CHO/IR in well-trained normal weight, overweight and obese pregnant women with type 1 diabetes across early, middle, and late pregnancy.

## Research Design and Methods

From 2006 to 2012, 101 pregnant Caucasian women with type 1 diabetes were followed in four Italian centers dedicated to the management of diabetes in pregnancy (7). We studied the 86 women who had more than one pregnancy outcome available in Electronic Medical Record (EMR). The four centers shared the same clinical management protocol for type 1 diabetic women in pregnancy and used the same EMR. Pre-gestational counseling and pregnancy management were implemented according to the Italian “Diabetes and Pregnancy Study Group” guidelines (17), in line with the American Diabetes Association (ADA) recommendations (18) and the Italian Standards of Care of Diabetes Mellitus (19). Glycemic targets were set out according to ADA guidelines (20). At the time, the recommended glycemic targets were ≤95 mg/dl at fasting, ≤140 mg/dl 1 h after the meal and ≤120 mg/dl 2 h after the meal.

HbA1c was checked at the first visit and monitored monthly, with the purpose of achieving values <6.0% (42 mmol/mol).

At the first visit all patients were trained on carbohydrate counting and on how to achieve and maintain strict metabolic control, with monthly refresher training sessions. Moreover, they received dietary recommendations with special reference to caloric intake and glycemic index, according to Italian Recommendations (21). The daily caloric intake, as well as the recommended weight gain during pregnancy, was prescribed in relation to pre-gestational body weight, in accordance with IOM (Institute of Medicine and National Research Council) recommendations (22).

The diet provided 45–50% carbohydrates, 20% proteins, and 30–35% fats, divided between breakfast (10–15%), lunch (20–30%), dinner (30–40%) and three snacks (5–10%; mid-morning, mid-afternoon, and before bedtime). A caloric surplus was assigned in the second (340 kcal) and third (450 kcal) trimester of pregnancy to ensure adequate energy reserves and normal fetal growth (21). The diet was verified through a self-monitoring diary where women recorded pre-prandial and 1-hour post-prandial blood glucose levels (4–8 measurements/day), meal composition, CHO grams of each meal, and insulin units needed. Total daily insulin requirements, basal and bolus units, were expressed both as absolute value and as IU/kg (23, 24).

All women reported GAD65 Ab titer positivity at the diabetes diagnosis.

Twenty-eight patients (32.6%) switched to CSII during pregnancy (at 11 ± 2.8 weeks of gestation), and the remaining patients used CSII therapy before conception.

Paradigm REAL-Time or Paradigm VEO (Medtronic Inc); Animas (West Chester, PA, IR 1200/2020) and ACCU-Chek Spirit (Roche Diagnostics) insulin pumps were used and only short-acting insulin analogs (lispro/aspart) were adopted.

The women were divided into three sub-groups according to their pre-gestational BMI (normal weight, overweight, and obese).

### Insulin Requirements and CHO/IR Calculation

Insulin requirements were expressed both as total I.U. per day and I.U. per body weight kg, as it currently happens in clinical practice/trials, giving information in women with different pregestational BMI and recommended weight gain.

The women measured CHO/IR daily at each meal by dividing the grams of CHO in the meal by insulin units needed. As the purpose of our study was to identify the CHO/IR able to reach the glycemic goals, we considered CHO/IR when the glycemic values were at target only.

In particular, the measured CHO/IRs were included in the analysis when the Fasting Capillary Blood Glucose (FCBG) ranged from 70 to 90 (acceptable up to 100 mg/dl), 1-h post-prandial BG values ranged from 100 to 130 (acceptable up to 140 mg/dl) and when CHO grams and insulin dose were correctly reported and congruent with medical prescription. During follow-up visits, the CHO/IR setting was verified and updated if necessary.

CHO/IR values were collected from patients’ diaries only. We could not use data from management system downloads as at that time only 26.7% of the women used sensors.

We only considered blood glucose levels written in the diaries and verified on a glucose meter.

The difference between CHO/IR at three different stages of pregnancy (Early:13–14^th^ g.w.; Middle: 27–28^th^ g.w.; Late: 33–35^th^ g.w.) was expressed as “Delta CHO/IR.”

The correction factor was calculated on the basis of the current 1,800 rule (25).

### Informed Consent

In agreement with our clinical practice, at the time of the first visit a written informed consent related to the use of clinical data (anonymously) was obtained from all women attending our outpatient offices.

### Statistical Analysis

All statistical analyses were performed using SPSS statistics version 20.0 (SPSS, Chicago, IL). Data are shown as median (25^th^ and 75^th^ percentiles) or mean (SD) according to distribution and as numbers (percentage). Kruskal–Wallis or ANOVA/Fisher tests were used to compare groups according to data distribution. Wilcoxon signed-rank and Friedman tests were used to analyze data longitudinally. Spearman correlation, univariate and multiple linear regression analysis were used to investigate relationships between variables. A p ≤ 0.05 was considered significant.

## Results

We enrolled 86 women with type 1 diabetes, aged 33.2 ± 5.2 yrs, disease duration 14 yrs (9–21) periconceptional HbA1C 52 (47–62) mmol/mol, BMI 24.4 (22.2–27.1) kg/m^2^, and weight gain at 33–35 g.w. of 14 (9.8–18) kg.

The patients were divided into three groups according to their pre-gestational BMI: 45 normal weight, 31 overweight and 10 obese women whose main clinical characteristics are shown in **Table 1**.

**Table 1:** Main clinical characteristics.

	Normal weight n. 45	Overweight n. 31	Obese n. 10	p-value
Age ± SD (yr)	33.2 ± 5.2	29.5 ± 5.5	31.7 ± 6.3	ns
Pre-gestational BMI (kg/m^2^)	22 (20.4–23)	26.7 (25.7–28.3)	34.4 (31.9–37.2)	ns
Disease duration (yr)	13 (6.5–21)	15.6 (10.2–19.5)	14 (11–22.7)	ns
HbA1c (mmol/mol)	49.7 (43.7-59.9)	49.2 (45-60)	49.5 (38.8-58)	ns

At 33–35 g.w., obese women gained less weight than the normal weight women (see **Table 3**) (Bonferroni, p = 0.015). The same trend was observed with respect to overweight women, without obtaining a statistical significance.

The main maternal-neonatal outcomes are reported in **Table 2**. No ketoacidosis episodes or severe hypoglycemia (requiring assistance of another person) were reported.

**Table 2:** Pregnancy Outcomes.

	Normal weight% (n)	Over-weight% (n)	Obese% (n)	Tot% (n)	p-value
CS	90 (36/40)	92.6 (25/27)	100% (10/10)	92.2 (71/77)	ns
Macrosomia	26.2 (11/42)	25 (7/28)	0 (0/9)	22.8 (18/79)	ns
LGA (>90th centile)	34.1 (14/41)	33.3 (9/27)	11.1 (1/9)	31.1 (24/77)	ns
Preterm birth (<34 g.w.)	0 (0/41)	0 (0/28)	40 (4/10)	5.1 (4/79)	0.001
Preterm birth (<37 g.w.)	24.4 (10/41)	28.6 (8/28)	50.0 (5/10)	29.1 (23/79)	ns
hypoglycaemia	15.8 (6/38)	25.9 (7/27)	22.2 (2/9)	20.3 (15/74)	ns
hypocalcemia	0 (0/38)	1 (1/27)	0 (0/9)	1.3 (1/74)	ns
Jaundice	13.1 (5/38)	29.6 (8/27)	11.1 (1/9)	18.9 (14/74)	ns
Female	46.3 (19/41)	46.4 (13/28)	20.0 (2/10)	43.0 (34/79)	ns

CS, Cesarean Section.

### Insulin Requirements and Glucose Control

Glycemic goals were generally reached before and after meals (see **Table 1 Supplementary file**).

#### Insulin Requirements: Longitudinal Trend in Each Group

In the normal weight women, insulin requirements, however expressed (24 h, basal or bolus total insulin and unit/kg, basal or bolus), significantly increased in late pregnancy (p < 0.0001). A similar trend was observed in the overweight women (<0.001; <0.001; 0.002, and 0.0006, respectively).

In the obese women, the insulin requirements, however expressed, did not change significantly during pregnancy (total insulin p = 0.14 and UI/kg, p = 0.1; total boluses p = 0.2, boluses/kg p = 0.4; total basal p = 0.1; basal/kg p = 0.27). Full data are reported in **Table 3**.

**Table 3:** Insulin requirement and weight gain: comparison among the three groups.

	EARLY 13–14^th^ g.w.					MIDDLE 27–28^th^ g.w.					LATE 33–35^th^ g.w.				
BMI	Normal(n = 38)	Over(n = 29)	Obese(n = 10)	p-value*	p-value**	Normal(n = 45)	Over(n = 31)	Obese(n = 10)	p–value*	p–value**	Normal(n = 45)	Over(n = 31)	Obese(n = 10)	p–value*	p–value**
**Weight Gain Kg**				0.0048	N *vs* Ob.002 Ov *vs* Ob.005				0.01	N vs O.02				0.04	N vs Ob.015
Median	3.1	2.25	−1.25			11	7.3	6			14	13.7	8.0		
25^th^–75^th^ Percentile	2–5	1–5.2	−3.1 −1.1			8–13	5–12	2.1–8.2			12–18.1	10–18.9	2.9–15		
**Insulin IU/24h**				0.0005	N vs Ob.001 N vs Ov.009 Ov vs Ob.01				0.004	N vs Ob.01				ns	ns
Median	33.30	41.15	53.5			38.63	46.20	58.08			49.8	56.45	62.64		
25^th^–75^th^ Percentile	23.6–51	23–67.8	46.4–59.2			22.5–97.6	39.1–75.9	38.6–73.7			32–101.6	26–102.3	38.9–83.2		
**Insulin IU/kg/24h**				ns	ns				ns	ns				ns	ns
Median	0.56	0.55	0.55			0.55	0.57	0.52			0.68	0.7	0.61		
25^th^–75^th^ Percentile	0.4–0.8	0.31–0.7	0.49–0.8			0.4–1.3	0.49–1.05	0.38–0.9			0.4–1.3	0.47–1.13	0.37–1.0		
**Basal IU/24h**				0.008	N vs Ob.01 N vs Ov.02				0.008	N vs Ob.005				0.02	ns
Median	17.2	24.1	30.88			21.13	25.35	33.58			24	30.45	38.92		
25^th^–75^th^Percentile	10.2–36.1	8.4–36.9	21.6–36			6.5–45.7	19.2–40.2	19.6–41.5			16–47	22–48.3	19.9–44.6		
**Basal IU/kg/24h**				ns	ns				ns	ns				ns	ns
Median	0.32	0.31	0.32			0.30	0.32	0.30			0.33	0.36	0.39		
25^th^–75^th^ Percentile	0–0.6	0.11–0.53	0.23–0.47			0.1–0.4	0.25–0.5	0.19–0.5			0.3–0.5	0.28–0.56	0.19–0.49		
**Bolus IU/24h**				0.0006	N vs Ob.01 N vs Ov.02				0.05	ns				ns	ns
Median	16.1	17.05	22.62			17.5	20.85	24.5			25.8	26	23.97		
25^th^–75^th^ Percentile	6.5–28.1	9–39.0	22.0–25			9.9–69	13.8–38	19–32.2			8.4–69	13.8–54	19–43.1		
**Bolus IU/kg/24h**				ns	ns				ns	ns				ns	ns
Median	0.23	0.23	0.26			0.26	0.26	0.22			0.35	0.31	0.22		
25^th^–75^th^ Percentile	0.1–0.5	0.11–0.53	0.2–0.33			0.1–0.9	0.17–0.5	0.19–0.4			0.1–0.9	0.17–0.56	0.18–0.5		

N, Normo–weight; Ov, Over–weight; Ob, Obese; ns, non-significant.

*Kruskal–Wallis, **Post hoc Analysis Bonferroni corrected p–values (S<0.016).

#### Insulin Requirements: Comparison Between Groups

In early pregnancy, insulin requirements increased according with BMI, being lower in the normal weight women and higher in the obese women (normal weight *vs* overweight p = 0.009; normal weight *vs* obese p = 0.001; see **Table 3**), while the insulin needs of the overweight women were in the middle (obese **vs** overweight p = 0.014; see **Table 2**). Basal and bolus insulin were in line with this trend (see **Table 3**).

These differences, although smaller, persisted in middle pregnancy (normal weight **vs** overweight p = 0.01; see **Table 3**), but were no longer evident in late pregnancy. Basal insulin requirements showed a similar trend (see **Table 3**), while differences in bolus doses were already lost by middle pregnancy (see **Table 3**).

When insulin requirements were adjusted for body weight (IU/kg), no difference was found in any phase of pregnancy (see **Table 3**).

#### Insulin Requirements: Relationships With BMI and Weight Gain in Whole Cohort

In early pregnancy, we found a significant correlation between pregestational BMI and insulin requirement expressed as total IU/day at each meal (p < 0.001), as confirmed by univariate linear regression analysis (*β* = 0.62; 95%CI (1–2); P < 0.001; R^2^ 0.3). In late pregnancy, neither pre-gestational BMI nor weight gain was independently related to the total insulin requirement increase recorded from early to late pregnancy.

### CHO/IR

#### CHO/IR: Longitudinal Trend in Each Group

At each meal, CHO/IR declined significantly throughout gestation in the normal (breakfast p = 0.01; lunch p = 0.03; dinner p = 0.0009) and overweight women (breakfast p = 0.001; lunch p = 0.006; dinner p < 0.0001) (see **Table 4**), while in the obese women it remained stable (see **Table 4**).

**Table 4:** CHO/I in normal weight, over–weight and obese women with type 1 diabetes across early, middle, and late pregnancy.

	Early 13–14 g.w.	Middle 27–28 g.w.	Late 33–35 g.w.	*p–value	**p–value	CHO/IR Delta
**Normal weight women (BMI<25)**						
CHO/I breakfast	13.3 (9.8–6.7)	7.5 (5.5–9.5)	6.2 (3.8–8.6)	0.01	EvsM 0.004 MvsL 0.04 EvsL 0.006	7.4 (0–11.4)
CHO/I lunch	12.4 (9–16)	10 (9–13.3)	7.9 (5.6–2.9)	0.03	EvsM 0.08 MvsL 0.002 EvsL 0.007	3.7 (0–6.4)
CHO/I dinner	14 (9.7–16.8)	10 (7.1–11.7)	6.9 (5.5–10)	0.0009	EvsM 0.1 MvsL 0.0004 EvsL 0.0005	6.1 (3.2–10.3)
**Over–weight women (BMI 25–29.99)**						
CHO/I breakfast	8.5 (7.1–12.6)	7.3 (5–9)	5.2 (4–8.1)	0.001	EvsM 0.08 MvsL 0.001 EvsL 0.0004	3.4 (1.5–5.4)
CHO/I lunch	11.3 (7.4–13.5)	8.5 (6.2–11)	7.3 (5.4–9.8)	0.006	EvsM 0.01 MvsL 0.01 EvsL 0.0006	3.1 (1.5–5.7)
CHO/I dinner	12.4 (8.9–16)	9.2 (7.5–12)	8.1 (6.7–13)	<0.0001	EvsM 0.02 MvsL 0.1 EvsL 0.001	1.7 (1–7.3)
**Obese women (BMI ≥30)**						
CHO/I breakfast	6.0 (5.0–7.9)	5,9 (4,7–8)	5.1 (4.1–7.4)	0.7	EvsM >0.9 MvsL 0.4 EvsL 0.4	1.4 (–3.4–3)
CHO/I lunch	7.8 (6.5–9.2)	7.1(4.8–9.3)	7.3 (4.1–10.7)	0.7	EvsM 0.5 MvsL 0.3 EvsL 0.7	0.7 (–3.4–3.5)
CHO/I dinner	9.3 (8.2–10.8)	7.3 (6.8–8.5)	7.2 (4.8–9.4)	0.2	EvsM 0.1 MvsL 0.5 EvsL 0.3	3 (–0.5–6.3)

Median (interquartile range).

Longitudinal analysis for each group: *p–value Friedman, **p–value Wilcoxon signed rank. E, Early, M, Middle, L, Late.

In addition, in order to assess if this overall CHO/IR decrease was a continuum process, we compared the CHO/IR changes happening between early and middle pregnancy or between middle and late pregnancy (see **Table 4**).

#### CHO/IR: Transversal Comparison Between Groups

In early pregnancy, the normal weight women had the highest CHO/IR at breakfast (*post-hoc* Bonferroni normal weight *vs* overweight p = 0.02; *vs* obese p = 0.01), even if, when compared with the overweight women, the difference was of borderline significance.

#### CHO/IR: Relationships With BMI and Weight Gain in Whole Cohort

In early pregnancy, we found a significant correlation between pregestational BMI and CHO/IR at each meal (p = 0.001), as confirmed by univariate linear regression analysis (beta −0.48; 95%CI (−0.78 to −0.21); P < 0.001; R^2^ 0.32).

In late pregnancy we found a significant correlation between pregestational BMI and CHO/IR change at breakfast and dinner (Spearman test, p = 0.03 and 0.048, respectively) as well as a correlation between CHO/IR change at breakfast and weight gain (p = 0.02). The significant relationship between BMI or weight gain and CHO/IR change at breakfast (univariate simple regression) was lost when both variables were included in a multiple regression analysis (see **Table 5**).

**Table 5:** BMI, weight gain and CHO/IR decrease at breakfast: univariate and multiple regression analysis.

	*β*	IC 95%	p	R^2^
Model 1. Dependent: Delta CHO/IR				
BMI	c0.341	−0.65 to -0.028	**0.033**	0.11
Model 2. Dependent: Delta CHO/IR				
Weight gain	0.38	0.05 to 0.55	**0.02**	0.14
Model 3. Multiple regression analysis. Dependent: Delta CHO/IR				
BMI	−0.2	−0.56 to 0.16	0.27	0.17
Weight gain	0.22	−0.07 to 0.5	0.14	

Delta CHO/IR is defined as the difference between CHO/IR in early pregnancy and CHO/IR in the late pregnancy.

The bold values of p provided are ≤0.05.

### Insulin Sensitivity (Correction Factor)

The calculated correction factor was higher in the normal weight women, lower in the obese women and intermediate in the overweight women in early (p = 0.0005) and middle (p = 0.0008) pregnancy. As pregnancy progressed, the calculated correction factor significantly decreased in the normal and overweight women only, while it remained unchanged in the obese women. As a consequence, in late pregnancy the three groups had a similar insulin sensitivity (see **Table 6**).

**Table 6:** Correction Factor across early, middle, and late pregnancy, in each group and between groups.

	Early 13–14 g.w.	Middle 27–28 g.w.	Late 33–35 g.w.	*p–value
BMI < 25 kg/m^2^	53.7 (47.6–62.8)	45.2 (34.9–53.6)	36.14 (29.0–44.7)	<0.0001
BMI 25–29.99 kg/m^2^	45.3 (38.4–51.1)	39.0 (29.3–47.1)	31.8 (23.5–40.7)	<0.0001
BMI ≥ 30 kg/m^2^	33.8 (30.7–37.7)	30.6 (24.9–36.5)	28.7 (23.8–32.4)	0.4
**p-value	0.0005	0.008	0.3	

Median (interquartile range) *p-value Friedman, **p-value Kruskal–Wallis.

## Discussion

In this study we found that insulin requirements, however, expressed (24-h total units or units/kg), increased from early to late pregnancy in the normal and overweight women by about 1.5 and 1.4 times, respectively. Similarly, CHO/IR decreased in both the normal and the overweight women.

In contrast, insulin needs did not change in the obese women, nor did CHO/IR (7).

However, considering the whole cohort the insulin increase rate was similar to that observed in some studies (26) but smaller than that observed in others (10, 27–29). These differences could have resulted from the different pregnancy time intervals considered and from different characteristics and lifestyles of the populations under investigation.

Analogously, the CHO/IR decrease was smaller in our study with respect of others such as that of Zagury et al. (30), who also found CHO/IRs higher than what we have observed. However, it should be considered that Zagury recruited women earlier and that the different CHO/IR pattern of the principal meals is similar to that of our study, with particular reference to breakfast. In fact, also our results prove that differences in insulin requirements and in CHO/IR at any stage of pregnancy are more evident at breakfast. This observation could result from the fact that breakfast is a high-carb meal and that the action of counter-regulating hormonal activity is maximal at the time of day when it is consumed.

We measured insulin requirements and CHO/IR from the 13–14^th^ g.w., as the women had reached a stable metabolic control (10) by then, until the 33–35^th^ g.w., as the majority of the obese women gave birth prematurely. On the other hand, there is evidence that obese women have an increased risk of pre-term deliveries, despite the pathophysiology of pre-term delivery not being well characterized (31).

During pregnancy, the insulin requirement increases with reference to the physiological increase in insulin resistance (32), and CHO/IR decreases (7, 26). Out-of-pregnancy fat mass is a well-known factor responsible for insulin resistance. Therefore, women with higher BMI are expected to become more and more insulin resistant, as partly supported by the relationship we found between pregestational BMI and insulin requirements in early pregnancy. On the contrary, in our obese women the total insulin requirement did not increase, and CHO/IR did not decrease. These findings are in line with those of Forbes et al. who found that, in contrast to lean women, HOMA-IR did not increase in obese non-diabetic women between mid and late gestation as well as between postpartum and early pregnancy. Moreover, the same authors, who studied the glucose endogenous production by clamp, found that insulin sensitivity in the liver improved during pregnancy in the severe obese women but was unchanged in lean ones (33).

We think that a relevant role is played by weight gain as we observed significant relationship between weight gain and CHO/IR change (univariate simple regression). Previous study showed that the changes in insulin sensitivity from the time before conception through early pregnancy were inversely correlated with the changes in maternal weight gain in lean women with normal and abnormal glucose tolerance (34).

Even if we did not report data from the beginning of pregnancy, we think that in obese women, the higher insulin resistance already documented in early pregnancy is mitigated by a lower weight increase—even weight loss in early pregnancy—compared to normal weight and/or overweight women in all pregnancy phases. This evidence may explain the differences in insulin requirements and CHO/IR among groups at baseline in contrast to late gestation, when the obese women achieved a lower weight gain. Moreover, the “quality” of weight gain could also have an impact on the insulin resistance increase rate. In fact, although we do not have data on the body composition and on the physical activity of our population, evidence shows that lean mass increases more in obese women than in normal weight women during pregnancy (14).

Since significant relationship between pregestational BMI or weight gain and CHO/IR change was lost in multiple regression analysis, we conclude that pregestational BMI and weight gain do not play an independent predictive role, but together contribute to CHO/IR change.

All the women received a dietary prescription as well as general training on the function of the glycemic index and macronutrient distribution in meals. Therefore, we think that a medical nutritional therapy appropriate in quantity and quality (22), likely plays a non-negligible role.

However, this hypothesis is not fully documented in our work since the patients’ eating habits were not investigated prior to the first visit. Moreover, the results of the study show that the obese pregnant women lost weight in the first trimester, despite the application of the abovementioned diet recommendations. Given that adherence to the diet was verified through the self-monitoring diary where women recorded the meal, the amount of carbohydrates, and the insulin used, we speculated that these findings can be derived from three factors: 1—MNT during pregnancy (19, 21) leads to a weight loss in obese women since they eat better and likely less than before pregnancy; 2—women during pregnancy are more motivated, strictly adhering to controlled diet plans; 3—emesis of early pregnancy could contribute to this weight loss, even if this phenomenon can affect all pregnant women irrespective of BMI.

These data obtained with old technologies were not influenced by an automated insulin suspension system and CHO/IR was selected only when pre- and post-prandial glucose levels were at target.

Finally, other variables not investigated in the present study could affect insulin requirements and CHO/IR. The first of these, fetal hyperinsulinemia, by lowering fetal glycemia increases the concentration gradient of glucose across the placenta, influencing the glucose flux from mother to fetus and reducing maternal glucose levels (35); then, post-prandial glucose control is impaired by slower glucose disposal (36) and slower insulin absorption as pregnancy advances (37); lastly, physical activity can have an impact on both weight gain and insulin requirements (38, 39)

The main study limitation is that this is a retrospective analysis sharing the same electronic database used in a previous study (7). Secondly the number of obese women is too small to draw definitive conclusions, so this finding should be confirmed in a larger population. Lastly, pregestational data are not available.

However, to date, few longitudinal data on CHO/IR in women with type 1 diabetes across early, middle, and late pregnancy are available and, to our knowledge, none with measured CHO/IR. This is the first study performed to assess CHO/IR in normal weight, overweight and obese pregnant women.

Deeper information might help in the management of pregnancy in type 1 diabetic women under insulin pump treatment, by predicting the CHO/IR trends during gestation. Therefore, a better practice in using technology may produce better outcomes.

## Conclusions

Pre-gestational BMI and weight gain contribute to determining the CHO/IR trend during pregnancy in pregnant type 1 diabetic Caucasian women under insulin pump treatment.

Although we cannot draw definitive conclusions as these retrospective findings need to be confirmed in a larger population, we think that these phenomena in obese women with type 1 diabetes in pregnancy deserve to be prospectively studied and considered in therapeutic algorithms of automated and manual insulin infusion systems in pregnancy.

## Data Availability Statement

The raw data supporting the conclusions of this article will be made available by the authors, without undue reservation.

## Ethics Statement

Ethical review and approval were not required for the study on human participants in accordance with the local legislation and institutional requirements. The patients/participants provided their written informed consent to participate in this study.

## Author Contributions

CF and AN: conceptualization, software, validation, writing— review and editing, and visualization. AN: methodology, supervision, and project administration. OB and CG: formal analysis. RF, MB, CS, CF, AN, NV, and CG: investigation and data curation. CF: resources and writing—original draft preparation. All authors contributed to the article and approved the submitted version.

## Conflict of Interest

The authors declare that the research was conducted in the absence of any commercial or financial relationships that could be construed as a potential conflict of interest.
