# Supplementary material for: Insulin Requirements and Carbohydrate to Insulin Ratio in Normal Weight, Overweight, and Obese Women With Type 1 Diabetes Under Pump Treatment During Pregnancy: A Lesson From Old Technologies
Source: Front Endocrinol (Lausanne). 2021 Feb 25;12:610877. doi: 10.3389/fendo.2021.610877 (PMC7959706; doi:10.3389/fendo.2021.610877)
Supplement: Supplementary file 1 [file Table_1.docx]

|  | **Normal weight** mean±SD | **Overweight**  mean±SD | **Obese**  mean±SD | **Tot**  mean±SD |
| --- | --- | --- | --- | --- |
| **EARLY** | | | | |
| Fasting BG mg/dl | 121±40 | 130,1±44 | 119,1±33 | 115,1±30,4 |
| 1h breakfast mg/dl | 131,7±35,3 | 110,4±35,8 | 139,3±85 | 146,9±51,4 |
| Before lunch mg/dl | 121,1±26 | 116,4±48,1 | 153±83,6 | 107,3±30,7 |
| 1h lunch mg/dl | 139,5±45,5 | 148,2±39,1 | 158,1±110 | 136,8±27,7 |
| Before dinner mg/dl | 127,4±45,5 | 145,2±29,9 | 142,8±37,8 | 121,1±28,1 |
| 1h dinner | 133,9±25,3 | 136,2±40,8 | 145,7±57,3 | 139,2±26,9 |
| **MIDDLE** | | | | |
| Fasting BG mg/dl | 119,3±31,1 | 110±19 | 107,8±28,4 | 115±30,4 |
| 1h breakfast mg/dl | 159,4±59,4 | 135,2±41,9 | 133,5±35 | 146,9±51,4 |
| Before lunch mg/dl | 112,9±31,5 | 104,7±36,5 | 105,5±22,3 | 107,3±30,7 |
| 1h lunch mg/dl | 132,6±28,1 | 156,2±19,8 | 115±18,7 | 136,8±27,7 |
| Before dinner mg/dl | 117,4±29,4 | 130,4±29,6 | 114,1±23,7 | 121,1±28,1 |
| 1h dinner | 143±26,4 | 140,3±16,4 | 129,2±42,4 | 139,2±27 |
| **LATE** | | | | |
| Fasting BG mg/dl | 111,1±25,4 | 114,1±32 | 106,2±28,8 | 115±30,4 |
| 1h breakfast mg/dl | 131±30 | 107,3±33,9 | 100,1±41,5 | 146,9±51,4 |
| Before lunch mg/dl | 114,8±39,6 | 106,3±20,1 | 101,7±61 | 107,3±30,7 |
| 1h lunch mg/dl | 119,6±24,2 | 129,3±23,5 | 126,2±17,1 | 136,8±27,7 |
| Before dinner mg/dl | 119,3±30,5 | 133,4±39,2 | 118±21,8 | 121,1±28,1 |
| 1h dinner | 147,4±48,9 | 154±40,3 | 144,9±28,8 | 139,2±26,9 |

**Table 1S. Capillary blood glucose levels before and 1 h after meals during early, middle and late pregnancy in normal weight, overweight, and obese women with type 1 diabetes.**
